# Predictors of acute undernutrition among children aged 6 to 36 months in east rural Ethiopia: a community based nested case - control study

**DOI:** 10.1186/1471-2431-14-91

**Published:** 2014-04-04

**Authors:** Gudina Egata, Yemane Berhane, Alemayehu Worku

**Affiliations:** 1 College of Health and Medical Sciences, Haramaya University, Harar, Ethiopia; 2 Addis Continental Institute of Public Health, Addis Ababa, Ethiopia; 3 School of Public Health, Addis Ababa University, Addis Ababa, Ethiopia

**Keywords:** Ethiopia, Undernutrition, Predictors, Under five children, Wasting

## Abstract

**Background:**

Child undernutrition is one of the major public health problems in the developing countries having a devastating effect on the lives of many children under five years of age. However, its causes are multitude and not uniformly understood enough across the various parts of the world and that a thorough understanding of these causes is required to design appropriate intervention. The objective of this study was to identify the predictors of acute child undernutrition in east rural Ethiopia.

**Methods:**

An unmatched community based nested case -control study was carried on 2199 (241 cases and 1958 controls) cohorts of children aged between 6–36 months with their respective mothers from July/August, 2010 to January/ February, 2011. The data were collected by using a pre-tested structured questionnaire and anthropometric measuring instruments which are recommended by UNICEF, after the standardization. Odds Ratio along with 95% confidence interval was estimated to identify determinants of wasting using the multivariable logistic regression.

**Results:**

Wasting was associated with poor [AOR (95% CI) = 1.49 (1.02, 2.20)] and middle [AOR (95% CI) = 1.52 (1.05, 2.20)] households’ socio-economic positions , individual based decision - making on the care or treatment of the ill child [AOR (95% CI) = 1.62 (1.20 ,2.20)], lack of maternal access to health facility [AOR (95% CI) = 1.56 (1.14, 2.20)], narrow birth interval [AOR (95% CI) = 1.65 (1.23, 2.20)], and non - exclusive breast feeding [AOR (95% CI) = 1.43 (1.05, 1.94)].

**Conclusions:**

Wasting was significantly associated with the households’ poverty, poor access to health services, lack of mutual decision – making on the care or treatment of their sick child between biological parents, closer birth interval, and poor exclusive breastfeeding practice. Thus, an organized effort should be made at all levels to improve infant and young child feeding , health services, child birth spacing behavior, and exclusive breastfeeding practice of the poor rural population particularly mothers to curb the problems of child undernutrition.

## Background

Acute child undernutrition is one of the major public health problems in the developing world claiming the lives of many children under five years of age. In this setting, the problem is pervasive and about 55 to 60 million of these children are wasted [1-3]. The magnitude of wasting is substantial and persistent in the Sub-Saharan Africa (SSA) [4] including Ethiopia where many children under-five are suffering from the effects of child undernutrition [5-9]. Evidence showed that child undernutrition is responsible for 54% of the deaths of children under five years of age (nearly 11 million children) globally [10,11] and for 51% of the deaths of Ethiopian children in the same age category [6,7,12].

Malnutrition encompasses both undernutrition and overnutrition [10,13]. Undernutrition, which results from inadequate intake of energy and other important nutrients, is often used interchangeably with malnutrition in many literatures [10,14,15].

In cognizant of the consequences of child undernutrition, it is important to understand its risk factors at different levels in the given society as they are multitude and hierarchically interrelated and not uniformly understood across the various regions of the world. Thus, a thorough understanding of these factors is required for a better intervention. In this regard, the United Nations Children’s Emergency Fund (UNICEF), in its malnutrition conceptual framework and other related literatures, identified three major risk factors that could lead to child undernutrition, namely, basic or structural, underlying (behavioral), and immediate (biological) risk factors [14,16].

Globally different literatures revealed that household (socio-economic and demographic) factors such as household’s poverty and income [4,17-19], residence, occupation [20-22], education [20,22,23], maternal age [24], family size and violence [20,21,25], overcrowding [19], lack of exposure to mass media [20,22,26,27], and non – use of iodized salt [22,28] have influenced the occurrence of acute child undernutrition. Concomitantly, some behavioral or community factors including lack of maternal and child health services, of adequate and safe water supply, and of improved environmental sanitation play their role [22,29].

Moreover, maternal undernutrition [2,21,24,27], narrow birth interval [23], child related factors such as child’s gender [18], age [22,27,30,31], weight at birth [20,21,23,24,27], and hospitalization [1,22,27,32], improper delivery of child health services, and poor Infant and Young Child Feeding practices (IYCF) [19-22] have been identified as proximate (individual) level risk factors for child wasting. There are some evidence that wasting was not associated with any of the IYCF [31,33,34] and the household food security status [35-37]. However, these results indicate the complexity of the problems at hand.

Although there is persistently high magnitude of acute child undernutrition in Ethiopia, available studies do not provide sufficient evidence on its risk factors at all corners of the country. In these studies, it was reported that child wasting was associated with some household factors such as family income and rural residence while community level factors included only the poor household sanitary facilities. On the other hand, maternal undernutrition and child related factors such as gender, low birth weight, and lack of appropriate IYCF were identified as the individual level factors [6-9,12,38]. However, most of these surveys were conducted on less sufficient number of study participants and used cross-sectional designs which are not appropriate to identify risk factors. Thus, this study was conducted to identify predictors of acute undernutrition among children aged 6 to 36 months in the rural east Ethiopia.

## Methods

### Study setting

This study was conducted in Kersa Demographic Surveillance and Health Research Centre (KDS-HRC) of Haramaya University, east Ethiopia. There were 48,192 adults and 7, 198 children under five years of age, living in 10256 households of the Demographic Surveillance Site (DSS). Most of these adults were illiterate and farmers [39]. The DSS is located in Kersa District which was divided into two semi-urban and ten rural ‘kebeles (the smallest administrative units in Ethiopia), and has three climatic zones-low land, midland and highland. In the district, there were no hospital and ambulance service and the nearest hospital was 50 km from the research site. However, there were three health centers and ten community health posts in the DSS. Each community health post had two health extension or community health workers who provide basic primary health care services. The primary health care coverage of the district was 80% in 2010 [40,41].

Agriculture is the main livelihood of most of the population of the DSS. Crop production is basically on annual basis, except in few locations where it is biannual. Sorghum and maize are the common grains cultivated in the district. Some potato and other vegetables are also scarcely produced. Crops that are good for family subsistence often planted during the wet season (June-August) and harvested in the dry season (December–February), while khat, a stimulant plant with amphetamine like effects, is predominantly produced as a cash crop. Polygamy is a very common in the area, and there are no profound cultural taboos related to IYCF [42].

### Study design and participants

A community based nested case-control study was conducted in the DSS from July/August 2010 to January/February 2011. A total of 2,352 mother–child pairs were enrolled into the follow - up study to determine the seasonal variation in the prevalence of acute child under-nutrition out of which 118 mother–child pairs did not complete their follow–up making a loss to follow up rate of 5%. However, among mother–child pairs who completed their follow-up, only 2,199 had plausible anthropometric measurements while the measurements of 35 children showed a flag sign beyond the standard range of values [43].

For the purpose of the follow-up study, the households in each kebele were enrolled into the follow-up using simple random sampling from the already available sampling frame of the KDS-HRC proportional to their estimated under five population size calculated from the total adult population of each kebele. In Ethiopia, the estimated proportion of the under five population is nearly 15% of the adult population [39].

Baseline survey was conducted on the randomly selected 2, 352 mother-child pairs from each selected household and the prevalence of acute child undernutrition was determined in wet season. The samples were drawn from the randomly selected households in each study kebele/village of the DSS proportional to the maximum sample size allocated for the study. If more than one 6 to 36 months of age children lived in the selected household, one child was selected by lottery method. The same mother–child pairs were then followed for 6–8 months. The population for this particular study consisted of sampled cases and controls of children 6 to 36 months of age and their mothers (mother–child pairs) who have been followed over the aforementioned period of time. The nutritional status of these children was again determined at the end line of the study in dry season when the available cases of under nutrition and the corresponding controls were identified. All cases and controls, who had credible anthropometric measurements within normal range of values for a weight-for-height z-score (WHZ) were included in the analysis regardless of the estimated sample size for this study to address other exposure variables of the study and increase the power of the study. In this study, cases of acute undernutrition (wasted children) were defined as the children whose WHZ - score was less than or equal to −2 standard deviation (SD) while the controls were those with greater than -2 SD score based on the existing evidence [14,44,45].

The sample size was computed using STATCALC application of Epi -Info 3.5.1 Statistical software with the following assumptions: proportion of illiteracy among the mothers of the controls to be 66.1% and of the cases 75 .0% [6], 5% type I error, 90.0% power of the study, control to case ratio of 4: 1 to detect an odds ratio of 1.54 [9] with a 20% contingency for none response. The exposure variable was educational attainment of women in the study setting [6]. Thus, the minimum sample size required for the study was 2,160 (540 cases and 1620 controls). However, as the number of cases identified at the end line of the study was less than the required sample size, all 2,199 (241 cases and 1.958 controls) children who had appropriate anthropometric data and their mothers were considered in this study.

### Measurements

The data were collected by using a structured and pretested interview questionnaire and anthropometric measurements. The questionnaire was initially prepared in English and then has been translated into the local language, *Afan Oromo*, by fluent speakers of both languages and again it was translated back into English to check its consistency. Data collectors and supervisors were obtained from KDS-HRC and the surrounding community. Both categories received intensive training for one week on the questionnaire and interviewing and anthropometric measurement techniques .Data collectors were paired during the data collection to ensure quality of the data. Anthropometric measurements were been taken after the proper training and standardizing procedures. A UNICEF recommended measuring and weighing instruments were used to collect the anthropometric data. Children below 24 months of age were measured in a recumbent position, while standing height was measured for those who were 24 months and older. Anthropometric measurements were taken twice and a difference of 100 gram in weight and 0.1 cm in length was accepted as normal. However, repeated measurement was carried out upon significantly larger differences [46]. Children were also assessed for the presence or absence of edema of the feet. Anthropometric data were calculated by using WHO Anthro2010 software and WHZ- scores were also been generated based on the WHO child growth standards which was introduced recently in 2006 [44].

The outcome variable was the nutritional status of the children selected for the study. In this study, the risk factors of child undernutrition were examined in the context of conceptual framework that was adopted from UNICEF’s malnutrition conceptual framework. This was done by organizing the explanatory variables into basic (household), community (underlying), and proximate (individual) level risk factors. The household factors included parents’ education, decision making strategy if the child is ill, wealth index and food security status. Maternal access to health facility and tetanus toxoid vaccination during the last pregnancy were considered as community factors, while individual factors included child’s characteristics such as age, sex, utilization of separate feeding plate, and minimum dietary diversity that was eaten by the children 24 hours preceding the survey, maternal birth interval, and exclusive breastfeeding.

Decision-making strategy by biological parents about care of their sick child was categorized as ‘individually made decision’ and ‘commonly/jointly made decision. The former is coded 0 while the latter coded 1. Individually made decision’ is a decision made by either the father or the mother alone. Such a decision making trait would be common when both parents of the child are not living together or could not reach consensus even while living together. Similarly, the household’s socio-economic status (wealth index) was assessed by using 28 variables. These variables included income, possession of durable assets, and cooperative bank saving account, sanitation facilities, source of drinking water, and housing conditions [6,7]. Regarding this, Principal Component Analysis (PCA) was computed to determine the households’ socio- economic position or wealth index. The categorical variables were made dummy before initiating the analysis, but the ordinal ones were ordered from the least important to the most important one [47]. Finally, the households were grouped into three: poor, middle, and rich and coded 1, 2, and 3, respectively.

The food security status of the households was determined based on nine standard Household Food Insecurity Access Scale (HFIAS) questions that were developed for this purpose by Food and Nutrition Technical Assistance (FANTA) in 2007. The respondents were asked about the amount and variety of meal eaten, and the occurrence of food shortage for the household members, causing them not to eat the whole day or eat at night only, in the past four weeks preceding the survey [48]. All “Yes” responses were coded as’ one and “No” responses were coded as zero, and the responses were summed to produce an index of household food insecurity. The index had a high internal consistency (Cronbach’s alpha = 0.90) [49]. Later on, food secure households were coded “1” and food insecure ones “0” for further analysis.

Furthermore, maternal access to health facility was categorized as “yes” and coded “1” and as “no” and coded “0”. In this study, access to health care facility was based on a proxy report of mothers to their main source of health care services, and the report of key informants in the respective kebeles of the study setting. It was also indicated that according to the principle of primary health care, accessibility refers to the availability of health care facilities for the clients within 10 km radius. It was also taken into account while determining access to health facility [41].

In this study, EBF was understood as feeding only breast milk without anything else for the first six months of life, with the exception of medicines for therapeutic purpose [50]. Minimum dietary diversity was defined as the proportion of children who were fed foods from 4 or more food items out of the seven major foods items within 24 hours preceding the survey [50]. It was categorized into two: ‘dietary intake from less than 4 major food items which was coded 0 and 4 or more major food items which was coded 1 .

### Ethical clearance

The study was cleared by the Ethical Review Committee of Haramaya University, College of Health and Medical Sciences, Ethiopia. Informed verbal and written consents were obtained from the parents/care givers of the children before the interview. Illiterate mothers consented by their thumb print after verbal consent.

### Data management and analysis

The data were double entered onto EPI- Data Version 3.1 by independent data clerk and were exported to SPSS Version 16. Multicollinearity was tested among the independent variables by using the Variance Inflation Factor (VIF) and the tolerance test. The result of the VIF ranges from 1.005–1.215 while the tolerance test was less than one, which was within the normal limit [51]. The factors that were supposed to interact were identified and entered together into the model in pairs and the interaction was checked at P < 0.05 significant level. Nevertheless, there was no interaction between the variables.

A bivariate analysis was performed on the independent variables and their proportions and crude odds ratio were computed against the outcome variable to identify the factors that are associated with acute child undernutrition. Then, three independent logistic regression models were constructed based on the knowledge of UNICEF’s malnutrition conceptual framework [16,52]. Each model was constructed based on the goodness of fit test and model coefficients tests. Thus, the Hosmer–Lemeshow goodness-of-fit and Omnibus tests of model coefficients with enter procedure were used to test for the model fitness. The variables that showed an association with the outcome variable at the bivariate analysis were put into the three hierarchical models and all the variables with p value ≤ 0.2 were entered into the final multivariable logistic regression model. However, the known determinants of child undernutrition such as a child’s sex and age, and the practice of EBF were entered into the model regardless of the p value. Odds ratio along with 95% confidence interval was estimated to assess the strength of the association and a P value < 0.05 was used to declare the statistical significance in the multivariable analysis.

## Results

A total of 2,199 child – mother pairs (241 cases and 1,958 controls) were included in the study. However, the results of some background variables were excluded for fifteen non-biological mothers due to the incomplete data. The mean (±SD) age of the cases was 29.0 (±9.05) months and 29.26 (±9.28) months for controls. The mean (±SD) age of the mothers of the cases was 30.15 (±6.02) years while it was 29.27 (±5.42) years for the mothers of the controls. Nearly equal proportion of the cases (89.2%) and the controls (88.2%) were from the rural residence. Most of the mothers of the cases (92.9%) were illiterate compared with the controls (86.8%). Similarly, most of the fathers of the cases (72.9%) were illiterate compared with the fathers of the controls (62.2%). Besides this, more cases (39.8%) were from poor households compared with the controls (32.5%), and most of the cases (53.9%) were the male children compared with the controls (49%) (Table 1).

**Table 1:** Socio – demographic and economic characteristics of the study participants by nutritional status, Kersa district, east Ethiopia, 2011

**Characteristics**	**Nutritional status**	
	**Cases (%)**	**Controls (%)**
Sex of household head		
Female	5(2.1)	25(1.3)
Male	236(97.9)	1933(98.7)
Relationship with a child		
Biological mother	240(99.6)	1944(99.3)
Care giver	1(0.4)(	14(0.7)
Maternal age (years)		
15–34	55(22.9)	407 (20.9)
35- 49	185(77.1)	1537(79.1)
Mean age (± standard deviation)	30.15(± 6.02)	29.37(5.42)
Residence		
Rural	214(89.2)	1715(88.2)
Urban	26(10.8)	229(11.8)
Ethnicity		
Oromo	236( 98.3)	1907(98.1)
Amhara	4(1.7)	(36.1)
Tigray	-(−)	1(0.1)
Religion		
Muslim	236(98.3)	1908(98.1)
Orthodox christian	4(1.7)	35(1.8)
Protestant	-(−)	1(0.1)
Maternal education		
Illiterate	223(92.9%)	1687(86.8%)
Literate	17(7.1%)	257(13.2%)
Marital status		
Single	1(0.4)	5(0.3)
Married	236(97.9)	1921(98.1)
Others	4(1.7)	32(1.6)
Maternal occupation		
Farmers	236(98.3)	1888(97.1)
Others	4(1.7)	56(2.9)
Paternal education		
Illiterate	172(72.9%)	1194(62.2%)
literate	64(27.1%)	727(37.8%)
Paternal occupation		
Farmers	231(97.9)	1850(96.3)
Others	5(2.1)	71(3.7)
Decision - making strategy of the sick child		1
Individual	149(61.8%)	424(72.7)
Jointly/common	92(38.2%)	534(27.3%)
Household’s socio-economic position (SEP)		
Poor	96(39.8%)	637(32.5%)
Middle	84(34.9%)	649(33.1%)
Rich	61(25.3%)	672(33.1%)
Child’s gender		
Male	130(53.9%)	959(49.0%)
Female	111(46.1%)	999(51.0%)
Child’s age (months)(N		
6–23	68(28.2)	544(27.8)
24–35	107(44.4)	(799 40.8)
36- 48	66(27.4)	615(31.4)
Mean age (± standard deviation)	29.02(±9.05)	29.26(± 9.28)

In the bivariate analysis, children who had illiterate mothers [COR(95% CI) = 2.00 (1. 20, 3.33)] and fathers [COR(95% CI) =1.64 (1.21, 2.21)], families in the poor [COR(95% CI) = 1.66 (1.18, 2.33)] and middle [COR(95% CI) =1.43 (1.008, 2.02)] socio-economic status, and who do not practice joint decision-making on the care of the sick child [COR(95% CI) = 1.70 (1.25, 2.17)] were more likely to be acutely undernourished at household level.

Correspondingly, children of mothers who had no access to the health facilities [COR(95% CI) = 1.74 (1.32, 2.28)] and TT vaccination during their last pregnancy [COR(95% CI) = 2.0 (1.06, 1.83 )] were at risk of undernutrition.

Likewise, narrow birth interval [COR(95% CI) = 1.60 (1.20, 2.09)], lack of separate feeding plate for the child [COR(95% CI) = 1.40 (1.01, 1.89)], and feeding a child from less than four major food items within 24 hours preceding the survey [COR(95% CI) = 1.53 (1.17, 2.00)] were identified as individual level factors that are associated with children’s nutritional status (Table 2).

**Table 2:** Bivariate analysis of selected characteristics of the study participants, Kersa district, east Ethiopia, 2011

**Characteristics**	**Nutritional status**		**COR (95% CI)**	**P -value**
	**Cases (%)**	**Controls (%)**		
Maternal education				
Illiterate	223(92.9%)	1687(86.8%)	1.99(1.20, 3.33)	0.008
Literate	17(7.1%)	257(13.2%)	1	
Paternal education				
Illiterate	172(72.9%)	1194(62.2%)	1.64(1.21, 2.21)	0.001
literate	64(27.1%)	727(37.8%)	1	
Decision - making strategy of the sick child				
Individual based	92(38.2%)	534(27.3%)	1.65(1.25, 2.17)	0.000
Jointly/common	149(61.8%)	1424(72.7)	1	
Household’s wealth index				
Poor	96(39.8%)	637(32.5%)	1.66(1.18, 2.33)	0.003
Middle	84(34.9%)	649(33.1%)	1.43(1.008, 2.02)	0.045
Rich	61(25.3%)	672(33.1%)	1	
Households’ food security				
Food insecure	29(12.0%)	180(9.2%)	1.35(0.89, 2.05)	0.157
Food secure	212(88.0%)	1778(90.8%)	1	
Maternal access to health facility				
No	100(41.5%)	568(29.0%)	1.74(1.32, 2.28)	0.000
Yes	141(58.5%)	1390(71.0%)	1	
Maternal TT vaccination				
No	142(58.9%)	993(50.7%)	1.39(1.06, 1.83)	0.017
Yes	99(41.1%)	965(49.3%)	1	
Exclusive breast feeding				
No	87(36.1%)	651(33.2%)	1.13(0.86, 1.49)	0.377
Yes	154(63.9%)	1307(66.8%)	1	
Birth interval				
< 2 years	155(64.3%)	1041(53.2%)	1.59(1.20, 2.09)	0.001
≥ 2 years	86(35.7%)	917(46.8%)	1	
Child had separate feeding plate				
No	184(76.3%)	1371(70.0%)	1.38(1.01, 1.89)	0.042
Yes	57(23.7%)	587(30.0%)	1	
MDD 24 hours before the survey				
< 4 food items	123(51.0%)	793(40.5%)	1.53(1.17, 2.00)	0.002
≥ 4 food items	118(49.0%)	1165(59.5%)	1	
Child’s gender				
Male	130(53.9%)	959(49.0%)	1.22(0.93, 1.59)	0.146
Female	111(46.1%)	999(51.0%)	1	
Child’s age (months)				
6- 23	68(28.2)	544(27.8)	1.17(0.81, 1.67)	0.403
24-35	107(44.4)	799(40.8)	1.25(0.90, 1.73)	0.181
36- 59	66(27.4)	615(31.4)	1	

COR = crude odds ratio, CI = confidence interval, MDD = Minimum Dietary Diversity, P - values are based on results from logistic regression.

In the first block logistic regression model, it was found that the children’s nutritional status was significantly affected by household level factors such as poor [AOR (95% CI) = 1.60 (1.10, 2.30)] and middle [AOR (95% CI) = 1.47 (1.03, 2.10)] household’s socio-economic status and lack of parental joint decision- making strategy on the treatment of the sick child [AOR (95% CI) = 1.84 (1.40, 2.50)], and paternal education [AOR (95% CI) = 1.44 (1.04,2.00). In the second model, lack of maternal access to health facilities [AOR (95% CI) = 1.70 (1.30, 2.20)] was significantly associated with acute child undernutrition among the community factors while in the third model, having a narrow birth interval [AOR (95% CI) = 1.62 (1.22, 2.15) and less dietary consumption from major food items within 24 hours preceding the survey [AOR (95% CI) = 1.49 (1.13 1.96)] were significantly associated with acute child undernutrition among the individual level factors (Table 3).

**Table 3:** Household, community, and individual level predictors of acute under nutrition among children 6 – 36 months, Kersa district, east Ethiopia

**Characteristics**	**Model I AOR (95% CI)**	**Model II AOR (95% CI)**	**Model III AOR (95% CI)**	**Final model AOR (95% CI)**
**Household (basic) factors**				
Maternal education				
Illiterate	1.59(0.93, 2.73)			1.42(0.82. 2.46)
Literate	1			1
Paternal education				
Illiterate	1.44(1.04, 2.00)*			1.38(0.99, 1.92)
Literate	1			1
Decision - making strategy of the sick child				
Individual based	1.84(1.40, 2.50)**			1.62(1.20, 2..20)**
Jointly/common	1			1
Households’ wealth index				
Poor	1.60(1.10, 2.30)*			1.49(1.02, 2.20)*
Middle	1.47(1.03 ,2.10)*			1.52(1.05, 2.20)*
Rich	1			1
Household food security				
Food insecure	1.39(0.90,2.14)			1,49(0.95, 2.34)
Food secure	1			1
**Community (underlying) factors**				
Maternal access to health facility				
No		1.70(1.30, 2.20)**		1.56(1.14, 2.20 )**
Yes		1		1
Maternal TT vaccination				
No		1.28(0.97, 1.68)		1.18(0.88, 1.58)
Yes		1		1
**Individual (proximate) factors**				
Exclusive Breastfeeding				
No			1,22 ( 0.92, 163)	1.43(1.05 , 1.9 4)*
Yes			1	1
Birth interval				
<2 years			1.62(1.22, 2.20 )**	1.65 (1.23, 2.20)**
≥ 2 years			1	1
Child had separate feeding plate				
No			1.29(0.93, 1.79)	1.13 (0.81,1.58)
Yes			1	1
MDD 24 hours before the survey				
< 4 food items			1.49((1.13, 1..96)*	1.25(0.93, 1.69)
≥ 4 food items			1	1
Child’s gender				
Male			1.19(0.91, 1.57)	1.19(0.89, 1.56)
Female			1	1
Child’s age (months)				
6- 23			1.15(0.80, 1.65)	1.13(0.78, 1.64)
24-35			1.29(0.93, 1.79)	1.36(0.97, 1.90)
36-60			1	1

* = p < 0.05, ** = P < 0.001, AOR = Adjusted Odds Ratio.

In the final multivariable model, children from households with poor [AOR (95% CI) =1.49 (1.02, 2.20)] and middle [AOR (95% CI) =1.52 (1.05, 2.20)] socio-economic status were nearly twice at increased risk of wasting. Similarly children whose parents did not make joint decision on the treatment of the sick child [AOR (95% CI ) = 1.62 (1.20, 2.20)], mothers lack access to health facilities [AOR (95% CI) = 156 (1.14, 2.20] and have narrow birth interval [AOR (95% CI) = 1.65 (1.23, 2.20)] were nearly twice more likely to be wasted. Non - exclusive breastfeeding [AOR (95% CI) = 1.43 (1.05, 1.94)] was also significantly associated with child wasting (Table 3).

## Discussion

In this study, acute undernutrition among the cohort of children, was associated with poor and middle households socio-economic positions, individually made decision strategy on the treatment/care of the sick child, lack of maternal access to health facilities, narrow birth interval, and non-exclusive breast feeding.

Among household factors, the households’ socio-economic status and lack of joint decision-making strategy on the care or treatment of the sick child have significantly affected children’s nutritional status. Based on the extensively reviewed literatures, in this study the role of parental decision making pattern was found to be the first of its kind in affecting acute child undernutrition. The odds of acute undernutrition were higher among children from families who made decision individually on the care or treatment of their sick children compared with the children whose families have made decision jointly on the issue. This could be explained by the fact that the provision of joint care by biological parents requires joint decision on the care or treatment of their children in order to improve children’s nutritional status. Such decision might also require women’s autonomy to participate in the decision making process of the household equally with the men.

Children from families in poor and middle socio-economic positions were more likely to be undernourished than their counterparts. This finding is comparable with the evidence from other similar studies conducted in low- income countries including Ethiopia [4,7,8,17,22] and indicates that wasting disproportionately affects the poor households.

In considering community level factors, only lack of maternal access to health service facilities significantly affected children’s nutritional status. In this study, these factors were considered as community factors as they were associated with behavioural attributes of the majority of the rural mothers in the studied community. The odds of exposure to the risks of acute undernutrition were higher among the children whose mothers had no access to the health service facilities than their counterparts. This is similar to the findings of other studies in which the expansion of healthcare infrastructures have significantly reduced the risk of child undernutrition [22,29]. In this study, access to health care facilities was estimated based on the proxy report of mothers which was based on mother’s main source of health care services and the report of key informants in the study community. According to the principle of primary health care, accessibility was referred to as the availability of health care facilities for the clients within 10 kilometers radius [41].

Among the individual level factors, exclusive breastfeeding and narrow birth interval were significantly associated with children’s nutritional status. The odds of acute undernutrition were higher among the children of gravid mothers whose birth interval was less than two years between the index-child and his or her older one compared to that of mothers whose interval was reported to be greater. This finding was in agreement with evidence from similar studies in which subsequent births led to child undernutrition [23]. This might be due to the short duration of breastfeeding which could result in inadequate intake of breast milk nutrients by the older child increasing the risk of undernutrition.

Non-exclusively breastfed children were more likely to be undernourished than their exclusively breastfed counterparts. This finding is consistent with similar studies conducted in low-income countries including Ethiopia [20,21,38]. This could be due to lack of essential nutrients from the breast milk during the first six months of life and later. These nutrients are known to prevent disease transmission by improving children’s immunity status and through interruption of infection-malnutrition cycle. This in turn improves child survival, growth, and development and prevents the sequel of undernutrition in later life [53,54].

Unlike other similar studies, among the basic (household) risk factors of child undernutrition, maternal education turned out to be insignificant in this study [20,22,23]. However, it was observed that paternal education had the marginal effect on the nutritional status of children. Lack of association between parental education and children’s nutritional status might be attributed to the overall literacy status of the study setting in that the majority of the parents involved in the study were illiterate. In the same line, none of the child related individual level risk factors were significantly associated with acute child undernutrition in contrast with the findings of other similar studies [18,20-23,26,27].

This study has strengths. One of its strengths is that it has used adequate sample size with initial random selection of the study participants from KDS -HRC data base by using a sampling frame to minimize a selection bias. The other is that in a nested case-control study recall and selection biases, which are common problems in standard case–control studies, can be avoided because exposure assessment did not require contact with study participants [55].

However, this study could have the following limitations. One is that the nutritional surveys are prone to technical error of anthropometric measurement (TEM), which could result in misclassification of children’s nutritional status. The study‘s findings could also be affected by recall and interviewer bias due to the retrospective tracking of information beyond the advantages of a nested case–control study. However, due attention was given to the study procedures, including the process of training the research team, standardization of anthropometric measurements , and a close supervision throughout the field activities to minimize the expected biases.

## Conclusions and recommendations

In sum, wasting was related to the households’ poverty, poor access to health services, lack of mutual decision – making on the care or treatment of their sick child between biological parents, closer birth interval, and non-exclusive breastfeeding practice. Thus, an organized effort should be made at all levels to improve infant and young child feeding, health services, child birth spacing behavior, and exclusive breastfeeding practice of the poor rural population particularly mothers to curb the problems of child undernutrition.

## Competing interests

The authors declare that they have no competing interests.

## Authors’ contributions

GE participated in the design of the study, performed the data collection, and the statistical analysis and served as the lead author of the manuscript. YB participated in the design of the study and contributed to the finalization of the manuscript. AW participated in the design of the study and helped perform the statistical analysis and as well participated in finalizing the manuscript. All authors read and approved the final manuscript.

## Authors’ information

GE is a lecturer in the Department of Public Health at Haramaya University, Ethiopia. He also has supervised many masters and under - graduate students. YB is a senior professor of epidemiology and public health and director of the Addis Continental Institute of Public Health, Ethiopia. He has been teaching several courses in public health including, epidemiology and research methodology in various Universities. He has also supervised many masters and doctoral students. He has more than 100 publications in national and international journals. AW is associate professor at Addis Ababa University, Ethiopia. He has been teaching biostatics and research methods in various Universities for many years. He has more than 40 publications in peer reviewed national and international journals.

## Pre-publication history

The pre-publication history for this paper can be accessed here:

http://www.biomedcentral.com/1471-2431/14/91/prepub

## References

[B1] Collins S Dent N Binns P Bahwere P Sadler K Hallam A Management of severe acute malnutrition in children Lancet 2006 368 9551 1992 2000 10.1016/S0140-6736(06)69443-9 17141707

[B2] Black RE Allen LH Bhutta ZA LE C e de Onis M Ezzati M Mathers C Rivera J Maternal and child undernutrition 1: maternal and child undernutrition: global and regional exposures and health consequences Lancet 2008 371 243 260 10.1016/S0140-6736(07)61690-0 18207566

[B3] Action Against Hunger Acute Malnutrition: A Preventable Pandemic 2009 International Network 247 West 37th Street, Floor 10 New York, NY 10018 212-967-7800 **Available**: [http://dd0jh6c2fb2ci.cloudfront.net/sites/default/files/publications/Acute_Malnutrition_A_Preventable_Pandemic_01.2009.pdf]

[B4] de Poel EV Hosseinpoor AR Speybroeck N Ourtia TV Vegab J Socioeconomic inequality in malnutrition in developing countries Bull World Health Organ 2008 86 282 291 10.2471/BLT.07.044800 18438517 PMC2647414

[B5] Central Statistical Agency [Ethiopia] Ethiopia Demographic and Health Survey Central Statistical Authority Addis Ababa 2001 Maryland, USA: Ethiopia 2000 ORC Macro Calverton

[B6] Central Statistical Agency [Ethiopia] and ORC Macro Ethiopia Demographic and Health Survey 2005 2006 Addis Ababa, Ethiopia and Calverton, Maryland, USA: Central Statistical Agency and ORC Macro

[B7] Central Statistical Agency [Ethiopia] and ICF International Ethiopia Demographic and Health Survey 2011 2012 Addis Ababa, Ethiopia and Calverton, Maryland, USA: Central Statistical Agency and ICF International

[B8] Edris M Assessment of nutritional status of preschool children ofGumbrit, North West Ethiopia Ethiop J Health Dev 2007 21 2 125 129

[B9] Medhin G Hanlon C Dewey M Alem A Tesfaye F Worku B Tomlinson M Prince M Prevalence and predictors of under- nutrition among infants aged six and twelve months in Butajira, Ethiopia: The P-MaMiE Birth Cohort BMC Public Health 2010 10 27 10.1186/1471-2458-10-27 20089144 PMC2826285

[B10] Blössner M de Onis M Malnutrition: Quantifying the Health Impact at National and Local Levels 2005 Geneva: World Health Organization

[B11] Collins S Treating severe acute malnutrition seriously Arch Dis Child 2007 92 453 461 10.1136/adc.2006.098327 17449529 PMC2083726

[B12] Macro International Inc Nutrition of Young Children and Women, Ethiopia 2005 2008 Calverton, Maryland, USA: Macro International Inc.

[B13] Benson T Bellete S Demese C Belachew T Gebremariam A Kumie A Hailemariam D Tesfaye F Genovese E An Assessment of the Causes of Malnutrition in Ethiopia: A Contribution to the Formulation of a National Nutrition Strategy for Ethiopia 2005 Washington, DC, USA: International Food Policy Research Institute

[B14] Maleta K Undernutrition Malawi Med J 2006 18 4 189 205 PMC3345626 27529011

[B15] Torpy JM Lynm C Glass RM Malnutrition in children JAMA 2004 292 5 648 10.1001/jama.292.5.648 15292091

[B16] UNICEF The State of the world’s Children: Malnutrition: Causes, Consequences and Solutions 1998 **Available**: [http://www.unicef.org/sowc98/sowc98.pdf]

[B17] Nandy S Irving M Gordon D Subramanian SV Davey Smith G Policy and practice poverty, child undernutrition and morbidity: new evidence from India Bull World Health Organ 2005 83 210 216 15798845 PMC2624218

[B18] Sharghi A Kamran A Faridan M Evaluating risk factors for protein-energy malnutrition in children under the age of six years: a case–control study from Iran Int J Gen Med 2011 4 607 611 21887115 10.2147/IJGM.S19499 PMC3160871

[B19] Odunayo IS Oyewole OA Risk factors for malnutrition among rural Nigerian children Asia Pac J Clin Nutr 2006 15 14 491 495 17077064

[B20] Hien NN Kam S Nutritional status and the characteristics related to malnutrition in children under five years of age in Nghean, Vietnam J Prev Med Public Health 2008 41 4 232 240 10.3961/jpmph.2008.41.4.232 18664729

[B21] Hien NN Hoa NN Nutritional status and determinants of malnutrition in children under three years of age in Nghean, Vietnam Pak J Nutr 2009 8 7 958 964

[B22] As O Oyekale TO Do mothers’ educational levels matter in child malnutrition and health outcomes in Gambia and Niger? Soc Sci 2009 4 1 118 127

[B23] Rayhan MI Hayat Khan MS Factors causing malnutrition among under five children in Bangladesh Pak J Nutr 2006 5 6 558 562

[B24] Vitolo MR Gama CM Bortolini GA Campagnolo P Drachler ML D.B Some risk factors associated with overweight, stunting and wasting among children under 5 years old J Pediatr (Rio J) 2008 84 3 251 257 10.2223/JPED.1776 18535734

[B25] Hasselmann MH Reichenheim ME Parental violence and the occurrence of severe and acute malnutrition in childhood Paediatr Perinat Epidemiol 2006 20 4 299 311 10.1111/j.1365-3016.2006.00735.x 16879502

[B26] Anderson AK Bignell W Winful S Soyiri I Steiner-Asiedu M Risk factors for malnutrition among children 5-years and younger in theAkuapim-north district in the Eastern Region of Ghana current research J Biol Sci 2010 2 3 183 188

[B27] Rahman A Chowdhury S Hossain D Acute malnutrition in Bangladeshi children: levels and determinants Asia Pac J Public Health 2009 XX X 1 9 10.1177/1010539509335399 19403548

[B28] Semba RD de Pee S Hess SY Sun K Sari M Martin WB Child malnutrition and mortality among families not utilizing adequately iodized salt in Indonesia Am J Clin Nutr 2008 87 438 444 18258636 10.1093/ajcn/87.2.438

[B29] MonteiroI CA D’Aquino BenicioI MH KonnoI SC Carolina FSA de LimaII ALL CondeI I Wolney L Causes for the decline in child under-nutrition in Brazil, 1996–2007 Rev Saude Publica 2009 43 1 1 8 10.1590/S0034-89102009000100001 19169574

[B30] Anderson AK Bignell W Winful S Soyiri I Steiner-Asiedu M Risk factors for malnutrition among children 5-years and younger in the akuapim-north district in the Eastern Region of Ghana Curr Res J Biol Sci 2010 2 3 183 188

[B31] Kumar D Goel NK Mittal PC Misra P Influence of infant-feeding practices on nutritional status of under-five children Indian J Pediatr 2006 73 5 417 421 10.1007/BF02758565 16741328

[B32] Wamani H Nordrehaug A Strøm A Peterson S Tumwine JK Tylleska RT Predictors of poor anthropometric status among children under 2 years of age in rural Uganda Public Health Nutr 2005 9 3 320 326 16684383 10.1079/phn2006854

[B33] Srivasteva N Sandhu A Index for measuring child feeding practices Indian J Pediatr 2007 74 4 363 368 10.1007/s12098-007-0061-7 17476081

[B34] Linda V Bahl R Martines J Penny M Bhandari N Kirkwood BR Group tWI-lVASS Use of new World Health Organization child growth standardsto assess how infant malnutrition relates to breastfeeding and mortality Bull World Health Organ 2010 88 39 48 10.2471/BLT.08.057901 20428352 PMC2802434

[B35] Psaki S Bhutta ZA Ahmed T Ahmed S Bessong P Islam M John S Kosek M Lima A Nesamvuni C Shrestha P Svensen E McGrath M Richard S Seidman J Caulfield L Miller M Checkley W Household food access and child malnutrition: results from the eight-country MAL-ED study Popul Health Metrics 2012 10 24 10.1186/1478-7954-10-24 PMC3584951 23237098

[B36] Osei A Pandey P Spiro D Nielson J Shrestha R Talukder Z Quinn V Haselow N Household food insecurity and nutritional status of children aged 6 to 23 months in Kailali District of Nepal Food Nutr Bull 2010 31 4 483 494

[B37] Hackett M Melgar-Quiñonez H Cecilia Álvarez M Household food insecurity associated with stunting and underweight among preschoolchildren in Antioquia, Colombia Rev Panam Salud Publica 2009 25 6 506 510 10.1590/S1020-49892009000600006 19695145

[B38] Amsalu S Tigabu Z Risk factors for severe acute malnutrition in children underthe age of five: a case–control study EthiopJHealth Dev 2008 22 1 21 25

[B39] Central Statiistical Authority [Ethiopia] Summary and Statistical Report of the 2007 Population and Housing Census. Population Size by age and sex 2008 Addis Ababa: Central Statistical Agency

[B40] Kersa DHO Health Service Coverage 2011 Ethiopia: Kersa District Health Office EasternHararge Oromia

[B41] FMOH Health Extension Program in Ethiopia 2007 Addis Ababa, Ethiopia: Profile Federal Ministry of Health

[B42] Kersa DAO Agricuture Extension program Kersa District Agricultural Office 2011 Oromia Ethiopia: Eastern hararge

[B43] Egata G Berhane Y Worku A Seasonal variation in the prevalence of acute child undernutrition among children under – five years of age in east rural Ethiopia: a longitudinal study BMC Public Health 2013 13 864 10.1186/1471-2458-13-864 24047474 PMC3851835

[B44] WHO WHO Child Growth Standards based on length/height, weight and age Acta Paediatr 2006 450 76 85 10.1111/j.1651-2227.2006.tb02378.x 16817681

[B45] Müller O Krawinkel M Malnutrition and health in developing countries CMAJ 2005 173 3 279 286 10.1503/cmaj.050342 16076825 PMC1180662

[B46] Cogill B Anthropometric Indicators Measurement Guide 2003 Washington, D.C: Food and Nutrition Technical AssistanceProject, Academy for Educational Development

[B47] Blakely T Hales S Woodward A Poverty: assessing the distribution of health risks by socioeconomic position at national and local levels 2004 Geneva: World Health Organization

[B48] Coates J Swindale A Bilinsky P Household Food Insecurity Access Scale (HFIAS) for easurement of Household Food Access: Indicator Guide(v. 3) 2007 Washington D.C: Food and NutritionTechnical Assistance Project, Academyfor Educational Development

[B49] Belachew T Hadley C Lindstrom D Gebremariam A Lachat C Kolster P Food insecurity, school absenteeism andeducational attainment of adolescents in JimmaZone Southwest Ethiopia: a longitudinal study Nutr J 2011 10 29 10.1186/1475-2891-10-29 21477343 PMC3090325

[B50] WHO Indicators for Assessing Infant and Young Child Feeding Practices 2008 Available: http://whqlibdoc.who.int/publications/2008/9789241596664_eng.pdf

[B51] Pan Y Jackson RT Ethnic difference in the relationship between acute inflammation and serum ferritin in US adult males Epidemiol Infect 2008 136 421 431 17376255 10.1017/S095026880700831X PMC2870810

[B52] Victora CG Huttly SR Fuchs SC Teresa A Olinto M The role of conceptual frameworks in epidemiological analysis: a hierarchical approach Int J Epidemiol 1997 26 1 224 227 10.1093/ije/26.1.224 9126524

[B53] WHO Learning From Large-Scale Community-Based Programmes To Improve Breastfeeding Practices 2008 Available: http://whqlibdoc.who.int/publications/2008/9789241597371_eng.pdf?ua=1

[B54] Al-Sahab B Lanes A Feldman M Tamim H Prevalence and predictors of 6-month exclusive breastfeeding among Canadian women: a national survey BMC Pediatr 2010 10 20 10.1186/1471-2431-10-20 20377899 PMC2858135

[B55] Langholz B Armitage P, Colton T Case–Control Study, Nested Encyclopedia of Biostatistics 2005 1 Second Edition 646 655

